# Study on risk factors (predisposing factors) for poor diabetes control during Hajj (1436/2015) in people with diabetes

**DOI:** 10.12669/pjms.325.11217

**Published:** 2016

**Authors:** Gulshad Hasan, Hanan Moabber, Arwa Alyamani, Ahmed Sayeed, Faisal Altatar

**Affiliations:** 1 Dr. Gulshad Hasan, FRCP (Principal Investigator) Division of Endocrinology, Department of Medicine, King Abdullah Medical City, Makkah, Saudi Arabia; 2 Dr. Hanan Moabber, Saudi Board – Internal Medicine Department of Medicine, King Abdullah Medical City, Makkah, Saudi Arabia; 3 Dr. Arwa Alyamani, Saudi & Arab Board – Internal Medicine Division of Endocrinology, Department of Medicine, King Abdullah Medical City, Makkah, Saudi Arabia; 4 Dr. Ahmed Sayeed, MD, MRCP, Department of Medicine, King Abdullah Medical City, Makkah, Saudi Arabia; 5 Dr. Faisal Altatar, Saudi & Arab Board – Internal Medicine Department of Medicine, King Abdullah Medical City, Makkah, Saudi Arabia

**Keywords:** Diabetes Mellitus, Predisposing factors, Hyperglycemia, Hajj

## Abstract

**Background and Objective:**

Each year millions of Muslims perform pilgrimage to Makkah, Saudi Arabia. It is particularly stressful during the peak five days, when all rituals have to be performed at specific periods of time at different sites. Poor diabetes control in people with diabetes predisposes to morbidity and increases risk of acute complications. We wanted to see how well their blood glucose control was before coming to Hajj and whether they were aware, about self management of Diabetes and what were reasons for hospital admissions.

**Method:**

We performed an observational prospective study, based on questionnaire. Sixty one patients were enrolled after taking informed consent. Patients included in the study were known or newly diagnosed diabetics who were admitted to KAMC between 1^st^ and 30^th^ Zil’Hajj.

**Results:**

Of the total 61 patients, 16 were newly diagnosed, (not known diabetic, before), while 45 were known diabetics. Among known diabetics, about 77% patients had poor diabetes control on admission, 72% did not bring glucometer, about 55% received diabetic education before coming to Makkah; 37% were doing SMBG occasionally and only 22% were aware that more frequent SMBG required during illness.

**Conclusions:**

Most people in our study population suffered from poor glycemic control before coming to Hajj. A significant number were unaware of their diagnosis. The most significant risk factor in our study was a lack of knowledge about self-management of diabetes and Hajj specific management.

## INTRODUCTION

Every year millions of Muslims travel to Makkah to perform Hajj. Hajj is the fifth pillar of Islam. It is a compulsory pilgrimage for all those who are able to bear its financial and physical burdens once in their lives. The rituals of Hajj take place approximately over one week from the 8^th^ to 13^th^ day of the month of Dhul Hijjah. For Hajjis (i.e. the people performing Hajj) this is a time of extraordinary physical and mental stress. In the context of diabetes, this means that Hajjis are at increased risk of both hyper and hypo glycaemia and increased morbidity and mortality from diabetes itself as well as inter current illness. Poor diabetes control before and during Hajj increases the risk of complications from diabetes, other pre-existing chronic conditions and inter current illness.^1^

By doing the study we wanted to find the reasons for poor diabetes control,^2^,^3^ during Hajj in Hajjis with diabetes admitted at King Abdullah Medical City, Makkah.

### Primary Objectives

To study the factors for poor diabetes control among Hajj pilgrims admitted to KAMC during Hajj and to find out about their self management practices.

### Secondary Objectives

To look for acute complications with which patients were admitted to hospital.

## METHODS

It is a prospective, cross-sectional, observational, pilot study based on questionnaire. It was conducted at King Abdullah Medical City (KAMC), Makkah, a large tertiary care referral center. Formal approval of the study was taken from Institution Review Board (IRB), Research Centre at KAMC. Data was collected by assistant consultants in Medicine, on a data collection Form. Consent was taken before asking questions and patients were explained the rationale behind this study. Confidentiality was maintained by not entering name in data entry Form. Patients were asked about diabetes control before coming to Hajj. Was diabetes education received before Hajj. Did they have glucometer with them, and whether they were doing self monitoring of blood glucose (SMBG) or not. Were diabetes medicines available to them and how was their compliance with prescribed medicines.

Random plasma glucose levels recorded at the time of admission were noted from the patients’ hospital record. Blood was taken later on for HbA1c. Patients who were not previously known to be diabetic but in whom the diagnosis was indicated due to clinical features or lab results were tested for random plasma glucose, fasting plasma glucose and HbA1c. They were diagnosed as being diabetic according to the following criteria.^4^

- HbA1c ≥ 6.5% OR
- Fasting plasma glucose ≥ 126 mg/dl OR
- 2 hour post-prandial plasma glucose ≥ 200 mg/dl during an OGTT OR
- Random plasma glucose ≥ 200 mg/dl in a patient with classic symptoms

Descriptive analysis of data was done after completion of study using IBM SPSS version 21.0.

### Inclusion Criteria

All adult diabetic patients, age 15 years and above, Hajjis only, admitted to King Abdullah Medical City Hospital for treatment during Murabata (i.e. high alert period lasting the first 2 weeks of Zil’Hajj) and two weeks after Murabata (i.e. from 1^st^ to 30^th^ Zil’Hajj).

### Exclusion criteria

Patients less than 15 years of age, patients with impaired mental capacity, patients with impaired level of consciousness who were unable to answer our questions and patients brought dead to the hospital.

## RESULTS

We enrolled a total of 61 patients. Of these 16 (26%) were newly diagnosed by us according to the aforementioned criteria, while 45 (74%) had pre-existing diabetes. Characteristics of our study population are shown in Table-I. Our findings in the patients who had pre-existing diabetes are as follows:

**Table-I:** Characteristics of patients included in to the study.

*Awareness of diagnosis*	
Aware	45 (74%)
Not aware (newly diagnosed)	16 (26%)
*Gender*	
Male	43 (70%)
Female	18 (30%)
*Age*	
21-40	3 (5%)
41-60	29 (47.5%)
61-80	29 (47.5%)
*Nationalities*	
Arabian	14 (23 %)
South East Asian	44 (72 %)
Others	3 (5 %)
*BMI^7^*	
< 25	20 (33%)
25 – 29.9	31 (51%)
> 30	10 (16%)
*Blood glucose on admission*	
< 200 mg/dl	27 (44%)
> 200 mg/dl	30 (49%)
Unknown	4 (1%)

**Fig.1:**
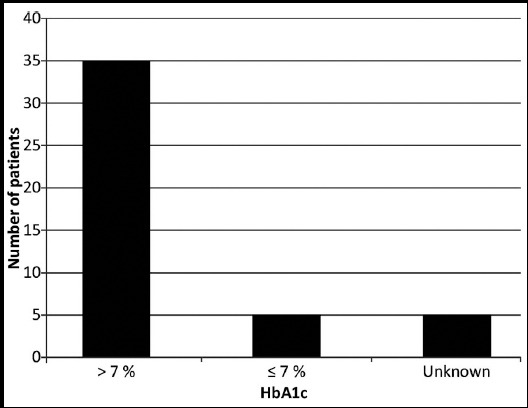
HbA1c of known diabetic patients.

**Fig.2:**
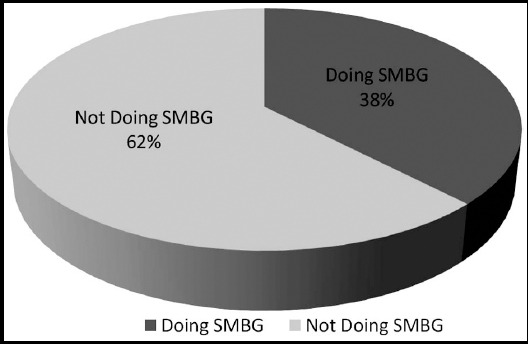
Patients performing SMBG (Among known Diabetics).

**Fig.3:**
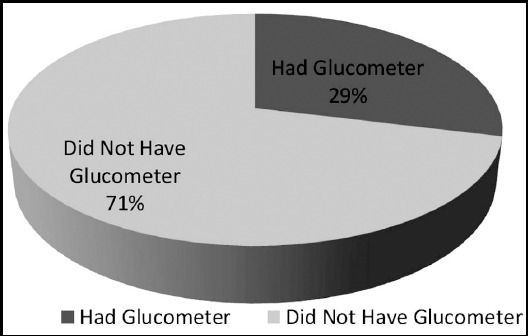
Patients having Glucometer (Among known Diabetics).

**Fig.4:**
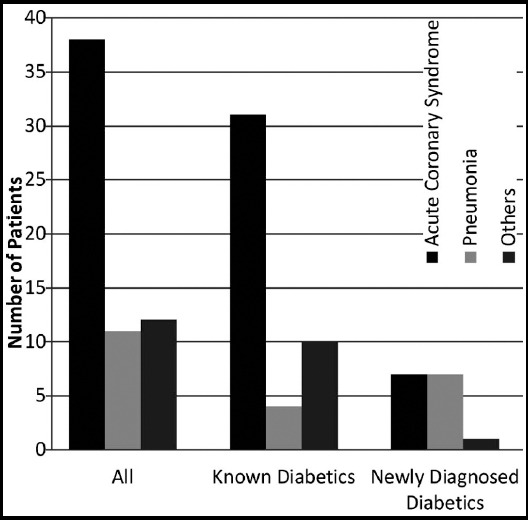
Reasons for Admission.

Thirty five patients (78%) had poor diabetes control at the time of admission (i.e. HbA1c of 7.1% or above) as shown in Fig.1.^5^,^6^ Twenty five patients (56%) received diabetic education before travelling for Hajj. Only 17 patients (38%) were doing SMBG (Fig.2) and only 10 patients (22%) were aware that more frequent SMBG is required during stress and illness. Thirty two patients (72%) did not bring a glucometer with them. Fig.3 Thirty seven patients (82%) had brought sufficient medication with them and among them, 35 (94%) patients were compliant with medication. The reason for admission of the patients is given below and shown in Fig-4. Among known diabetics the primary reason for admission was Acute Coronary Syndrome (31 patients) followed by pneumonia (4 patients). Stroke and COPD accounted for two patients each. We received one patient each of diabetic ketoacidosis, upper gastrointestinal hemorrhage, heat exhaustion, diabetic foot, atrial fibrillation and acute kidney injury.

Among newly diagnosed diabetics there were seven patients each of acute coronary syndrome and pneumonia, one patient of bronchial asthma and one of iatrogenic Cushing’s syndrome with fractured neck of femur and secondary diabetes because of prolonged use of topical steroid for more than two years for some dermatological condition.

### Additional Observations

Six out of ten patients admitted on Arafat day had stopped taking their medications. Two patients were taking herbal medications for control of blood glucose. Two patients had misconceptions that in Makkah there was no need to take medicines or restrict diet for blood glucose control.

## DISCUSSION

Our study shows that the majority of Hajj pilgrims admitted to KAMC during the month of Dhul Hijjah came from countries from southeast Asia which have relatively high rates of poverty and resultantly poor healthcare.^8^,^9^ This was reflected in the high number of Hajjis who did not receive any form of diabetic education. It was also reflected in the low numbers of pilgrims who had adequate control over their blood sugar levels as evidenced by an HbA1c of 7% or less. Most diabetic pilgrims were unaware of the need of increased SMBG during times of illness or stress^10^,^11^ which was evinced by the small percentage of pilgrims who brought glucometers with them and were performing SMBG. It also came out during our conversations with patients, that some of them did possess glucometer but did not feel the need to bring them along for Hajj.

It was also interesting to note that about 26% of patients were not aware of their diagnosis prior to their admission at KAMC. Had these people been screened for diabetes before travelling for Hajj and had received appropriate advice and management, it is possible they might have avoided hospital admission altogether or at the very least, be at reduced risk for increased morbidity and mortality associated with poorly controlled diabetes.^1^ This is especially true in the case of the patient with iatrogenic Cushing’s syndrome.

Our study also shows that there is a need to counter myths and misconceptions regarding the management of diabetes during Hajj. This is shown by the two patients we received who believed that there was no need to monitor blood glucose or take medications in Makkah and by the disproportionately high number of patients received on Arafat day who were aware of their diagnosis but had stopped taking their medication.

### Limitations of the study

Our study was limited to a relatively small patient population. The high prevalence of poorly controlled diabetes in our patient population as well as the lack of appropriate self management and diabetic education warrants a large, multi-centre study to determine the exact impact of these and other factors on morbidity and mortality of pilgrims during Hajj.

## CONCLUSION

Our study indicates that poor diabetes control is a serious problem among pilgrims and there are a variety of risk factors predisposing to it. Our study also found that there were a substantial number of patients who did not know that they were diabetic. This has led us to believe there is a need to screen all people coming for Hajj for Diabetes. Hajj specific health education needs to be introduced for people with diabetes and perhaps for other diseases as well.

### Recommendations:^12^

1. All Hajjis should be screened for diabetes with Fasting blood glucose and HBA1c.
2. They are advised to see their treating physician 3 to 4 months before travelling for Hajj.
3. They should receive Hajj focused Diabetes education before travelling for Hajj.
4. They should be provided with information leaflet (given below), regarding diet control, exercise, Self-monitoring of Blood glucose and medication compliance.
5. Each patient or at least each group should have medical or paramedical member with glucometer.

Information Leaflet for People with Diabetes Travelling for Hajj:

Kindly read this information if you are diabetic and travelling to perform Hajj:

1. See your doctor at least 3 to 4 months before travelling to Hajj
2. Receive education for self monitoring of blood glucose and what to do in case blood glucose is high or low.
3. Get advice about your diet as well. If possible, try to take same kind of diet you were taking before travelling for Hajj.
4. You should have laboratory investigations, including HBA1c (average reading for 3 months blood glucose) and renal function assessment, which helps if there is a need to adjust your medicines.
5. Blood pressure should be checked and treated to keep it within a target value. Good blood pressure control helps to improve blood glucose control.
6. Take medications regularly as prescribed by your doctor and remember to take food in time. Delays in taking your meals can cause your blood sugar levels to become dangerously low.
7. During Hajj, Monitor your blood glucose from time to time to keep an eye on your blood glucose.
8. If you get ill, then monitor your blood glucose at least 2 to 3 times a day and seek immediate medical help.
  Manifestations of high blood glucose include, feeling unwell, increased thirst, passing urine frequently. In addition, manifestations of infections, such as sore throat, fever, cough, burning while passing urine, etc.
9. You should keep a glucometer (with test strips, lancet and alcohol swabs) with you for Self Monitoring of blood glucose.
10. During Hajj, you may need to walk for long periods of time, that you may not be used to. During walking or exercise blood glucose may drop. If blood glucose goes too low then you can get manifestations of low blood glucose which include sweating, palpitations, dizziness, trembling hands, feeling cold, confusion and even loss of consciousness.
  Therefore keep with you something sweet to take, for example dates (tamar), sugar or juice. If blood glucose is low, 2-4 dates could be taken with water or 2 to 4 teaspoon full of sugar can be taken with water. Recheck your blood glucose after 15 minutes. Subsequently take something solid like a sandwich or your usual meal, so that blood glucose does not drop again.
11. Seek immediate medical help from nearest medical centre, if you are unwell. Your group leader should be aware of that.
